# Microbial Quality Assessment and Efficacy of Low-Cost Disinfectants on Fresh Fruits and Vegetables Collected from Urban Areas of Dhaka, Bangladesh

**DOI:** 10.3390/foods10061325

**Published:** 2021-06-09

**Authors:** Md. Mafizur Rahman, Md. Obyedul Kalam Azad, Jasim Uddain, Md. Adnan, Md. Chayan Ali, SK. Md. Jakaria Al-Mujahidy, Md. Zohurul Kadir Roni, Mohammed Saifur Rahman, Md. Jahirul Islam, Md. Hafizur Rahman, Ki Young Choi, Most Tahera Naznin

**Affiliations:** 1 Department of Biotechnology and Genetic Engineering, Faculty of Biological Sciences, Islamic University, Kushtia 7003, Bangladesh; mmrahman@btge.iu.ac.bd (M.M.R.); chayanali7@gmail.com (M.C.A.); jmujahidy@gmail.com (S.M.J.A.-M.); 2 Department of Bio-Health Convergence, College of Biomedical Science, Kangwon National University, Chuncheon 24341, Korea; azadokalam@gmail.com (M.O.K.A.); mdadnan1991.pharma@gmail.com (M.A.); jahirulislam213@gmail.com (M.J.I.); hafizknu94@gmail.com (M.H.R.); 3 Department of Horticulture, Sher-e-Bangla Agricultural University, Dhaka 1207, Bangladesh; uddain.jasim@gmail.com; 4 Tropical Agriculture Research Front, Japan International Research Center for Agricultural Sciences (JIRCAS), 1091-1 Maezato-Kawarabaru, Ishigaki, Okinawa 907-0002, Japan; ronisau@gmail.com; 5 Department of Biosystems and Technology, Swedish University of Agricultural Sciences, 75007 Alnarp, Sweden; morn0004@stud.slu.se; 6 Department of Controlled Agriculture, College of Agriculture and Life Sciences, Kangwon National University, Chuncheon 24341, Korea

**Keywords:** decontaminating agents, food safety, bacteria, human health

## Abstract

This study aimed to examine the total viable bacteria (TVBC); total coliform (TCC); fecal coliform (TFC); pathogenic *Pseudomonas* spp., *Staphylococcus aureus,* and total fungi (TF); and the effect of different low-cost disinfectants (sterile water, salt water, blanched, and vinegar) in decontamination of 12 types of fruit and 10 types of vegetables. In fruit samples, the lowest TVBC was enumerated at 3.18 ± 0.27 log CFU/g in Indian gooseberry and the highest at 6.47 ± 0.68 log CFU/g in guava. *Staphylococci* (2.04 ± 0.53–5.10 ± 0.02 log CFU/g), *Pseudomonas* (1.88 ± 0.03–5.38 ± 0.08 log CFU/g), and total fungi (2.60 ± 0.18–7.50 ± 0.15 log CFU/g) were found in all fruit samples; however, no *Salmonella* was detected in fruit samples. Similarly, the lowest TVBC recorded 5.67± 0.49 log CFU/g in cucumber and the highest 7.37 ± 0.06 log CFU/g in yard long bean. The *Staphylococci* (3.48 ± 0.13–4.81 ± 0.16 log CFU/g), *Pseudomonas* (3.57± 0.21– 4.75 ± 0.23 log CFU/g), TCC (1.85 ± 1.11–56.50 ± 37.14 MPN/g), TFC (1.76 ± 0.87– 3.78 ± 3.76 MPN/g), and TF (3.79 ± 0.18–4.40 ± 0.38 log CFU/g) were recorded in all vegetables samples, but no *Salmonella* was detected in yard long bean, pointed gourd, carrot, tomato, cucumber, or brinjal. However, vinegar showed the highest microbial load reduction of selected fruit and vegetables among the different treatments. With vinegar treatment, the highest reduction of TVBC (1.61-log) and TF (2.54-log) was observed for fruits, and TVBC (2.31-log) and TF (2.41-log) for vegetables. All the disinfectant treatments resulted in significant (*p* < 0.01) bacterial load reduction compared to control for the studied fruits and vegetable samples.

## 1. Introduction

Dhaka, the capital of Bangladesh, is one of the most densely populated fast-growing cities in the world [1]. Most of the fresh food in Dhaka comes from various rural areas of Bangladesh. Food grown in rural areas is highly contaminated due to a lack of adequate knowledge of food quality and safety [2]. There is a widespread practice of consuming fresh vegetables and fruit (FFV) in Bangladesh [3]. It is well known that the benefits of consuming FFV are related to the provision of healthy food constituents that play an important role in reducing the risk of certain non-contagious chronic diseases, such as cancer, cardiovascular, and neurodegenerative diseases [4,5,6]. However, the method of production of fresh food has a high possibility with potential sources of microbial contamination [7]. The different FFVs are consumed raw, thus posing a potential food safety problem due to different pathogenic microorganisms. Different factors, including the location of samples, growth conditions, collection season, and microbiological analysis method [8] can significantly vary the microbiological qualities and prevalence of pathogens in fresh produce.

There are three categories (satisfactory, acceptable, and not acceptable) of FFV samples in terms of the guidelines [9]. The TVBC contamination levels are satisfactory (<10^4^), acceptable (>10^4^–10^6^), and not acceptable (>10^6^) in terms of ready-to-eat salad vegetables [9]. Besides this, the CDC guidelines for the microbiological quality for ready-to-eat foods range from satisfactory to potentially hazardous. The unsatisfactory level of ready-to-eat foods is as follows the presence of *Salmonella*; *Staphylococci* (log 3 to 4); and fecal coliform (FC), especially *E. coli* (*n* > 3) presence (CDC, 2001). Moreover, the overall microbiological specification criteria of FFVs are mentioned in several guidelines [9,10,11,12]. The presence of *Salmonella*, *E. coli*, or a higher level of *Pseudomonas* and *Staphylococci* could be an indicator of overall higher risk of tested FFVs.

The consumption of contaminated fresh vegetables is connected to occurrences of human food poisoning [13,14,15]. Outbreaks of foodborne illness are caused by contamination of FFV, which is exposed to fecally infected manure fertilizers, irrigation with fecally contaminated water, and/or contaminated ice washing during handling and transport [16]. Several studies indicated that fecal coliform bacteria, i.e., *Escherichia coli*, *Shigella* spp., *Pseudomonas* spp., *Salmonella* spp., *Listeria monocytogenes*, and *Clostridium botulinum*, are common pathogenic microorganisms associated with food-borne illness. Recent outbreaks with pathogenic *E. coli* have been identified due to the consumption of fresh vegetables, such as carrots, radish sprouts, lettuce, alfalfa sprouts, spinach, grapes, and berries [17,18]. In another study, it was also found that the pathogenic serotype of fecal coliform *E. coli* O104:H4 was associated with the Shiga toxin-related hemolytic uremic syndrome in Germany [19], and *E. coli* O157:H7 infection in Japan [20] due to the consumption of sprouts and Chinese cabbage. In the USA, the average amount of FFVs are consumed at around 741 pounds per person annually, and over the last two decades, fresh fruit and vegetable consumption has increased at least by 25% [21,22]. In Europe, the consumption of salad vegetables has increased on average by 10% per annum [23]. Moreover, several outbreaks caused by food-borne pathogens have increased in worldwide, which are linked with consumption of raw fruit and vegetables [22,23,24,25,26].

The enteropathogenic *E. coli*, enterotoxigenic *E. coli,* and *Vibrio cholerae* are the predominant causative agents of food-borne linked outbreaks in Bangladesh, principally due to consumption of contaminated fresh produce [27,28,29]. More than one-third of children under five years of age in Bangladesh suffer from enteric diseases caused by contaminated food. As a result, an increase in the incidence of foodborne disease outbreaks associated with fresh fruit consumption has been observed in Bangladesh over the last two decades [28,29]. During post-harvest processing, frequent washing can completely remove soil and debris but not pathogenic microorganisms, which can cause cross-contamination of other foodstuffs, cookware, utensils, and so on [20].

Microbiological analysis is a valuable way of evaluating the emerging risk that concerns both the monitoring authorities and food consumers as well [30]. Disinfectant wash is essential to reduce fresh fruit and vegetable microbial loads [20]. There are many strategies, i.e., physical and chemical treatments, which have been studied to decontaminate fresh-cut fruit and vegetables. As a decontaminating agent, the chlorinated solution is used for fresh fruit and vegetables. Due to the low cost and ease of holding of chlorine, it is used mostly in liquid form [20,31]. Besides this, water supplemented with varying concentrations of organic acids, likely acetic acid and citric and sorbic acids, has been shown to reduce microbial populations on fruit and vegetables [32,33]. However, there are very few reports of microbial contamination of fresh-cut vegetables and fruit in Bangladesh [34,35,36]. There is a very limited amount of microbial quality evaluation research and investigation of effectiveness of low-cost disinfectants of fresh fruit and vegetables has been carried out in Dhaka City to the best of our knowledge.

The aim of this present study was therefore to determine the overall viable bacterial load of fresh fruit and vegetables collected from Dhaka City, Bangladesh, and to assess the effectiveness of low-cost disinfectants in the decontamination of fresh fruit and vegetables.

## 2. Materials and Methods

### 2.1. Sample Collection

**Figure 1:**
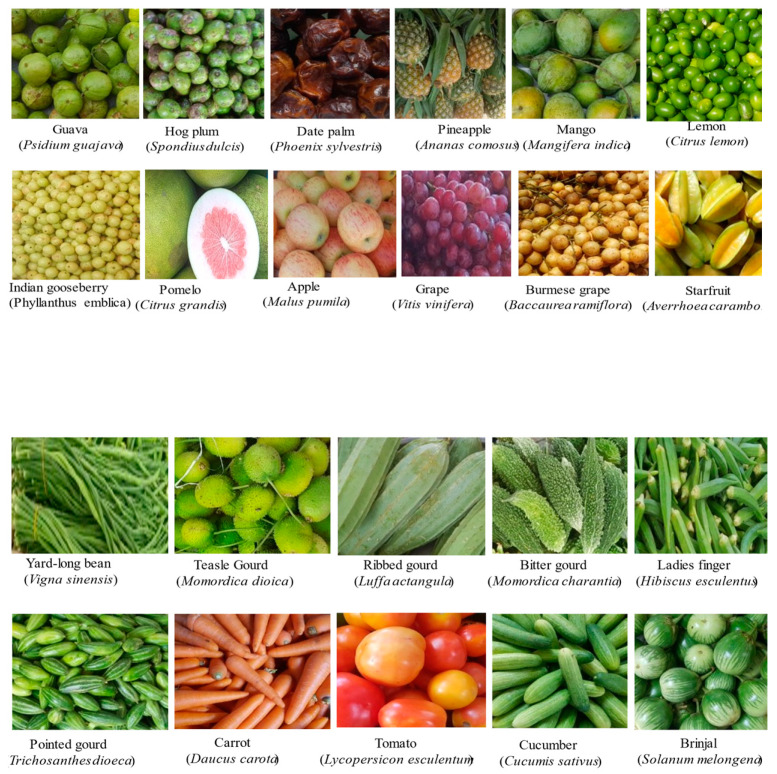
Fruit and vegetables collected from four different locations in Dhaka City.

A total of 88 fresh fruits and vegetables samples (FFV) comprising 12 types of fruit such as, guava (*Psidium guajava*), hog plum (*Spondius dulcis*), date palm (*Phoenix sylvestris*), pineapple (*Ananas comosus*), mango (*Mangifera indica*), lemon (*Citrus lemon*), Indian gooseberry (*Phyllanthus emblica*), pomelo (*Citrus grandis*), apple (*Malus pumila*), grape (*Vitis vinifera*), Burmese grapes (*Baccaurea ramiflora*), starfruit (*Averrhoea carambola*) and 10 types of vegetables such as, yard-long bean (*Vigna sinensis*), teasle gourd (*Momordica dioica*), ribbed gourd (*Luffa actangula*), bitter gourd (*Momordica charantia*), ladies finger (*Hibiscus esculentus*), pointed gourd (*Trichosanthes dioeca*), carrot (*Daucus carota*), tomato (*Lycopersicon esculentum*), cucumber (*Cucumis sativus*), and brinjal (*Solanum melongena*) were collected and kept in sterile ice bags from four different locations (Mohammadpur, Jatrabari, Uttara, and Gulshan) in Dhaka City (Figure 1). The microbiological tests were carried out in the food microbiological laboratory, Institute of Food Science and Technology (IFST), Bangladesh Council of Scientific and Industrial Research (BCSIR), Dhaka, Bangladesh.

### 2.2. Sample Processing

Twenty grams of each sample was aseptically mixed (1:10) with 180 mL Ringer solution [37] into a sterile conical flask. The sample was homogenized with a blender at 6000 rpm for 5–10 min. The samples were diluted six times (10^−1^–10^−6^) to reduce the concentration of microorganisms in the ringer solution. The number of colonies in an appropriate dilution was multiplied by the dilution factor to obtain the total viable bacteria count (TVBC), which was expressed as a mean colony-forming unit (CFU) per gram.

### 2.3. Microbiological Analysis of Fresh Fruit and Vegetables

A total of 0.1 mL of each decimal dilution was distributed over 20–25 mL of plate count agar (PCA) for the calculation of total viable bacteria (TVBC), and the plates were incubated at 37 °C for 24–48 h. Triplicate agar plates were counted between 30 and 300 colonies. A colony counter was used to count the colonies [3]. The samples were analyzed with the most probable number (MPN) technique to detect the total coliform and fecal coliform counts (TCC and TFC) [38]. Lauryl tryptose broth (LTB) and brilliant green lactose bile broth (BGLBB) were used for this test. As a presumptive measure, the LTB was used, and for the confirmatory and completed test, the BGLBB was used. The tubes with lauryl sulphate tryptose broth (LTB) were inoculated with (10^−1^, 10^−3^, 10^−6^) diluted samples for 48 h at 35 °C for the TCC and TFC count. In each broth, Durham tubes were inserted to observe the gas formation [39]. After 24 h of incubation, each set of positive tubes that displayed positive acid and gas were counted and calculated using the standard MPN table. The positive sample for the completed test was suspended in BGLBB medium at 1 mL and kept at 37 °C at 24–48 h, and the positive results for the tube were determined with the MPN table [39].

One milliliter of initial dilution (1:10) was transferred to the 9 mL tube of Ringer solution, which was prepared for 10^−2^ dilution for the enumeration of *Staphylococcus* spp. and *Pseudomonas* spp. The dilution was prepared until 10^−6^ in this way. One millilter of suspension was transferred for each decimal dilution for the isolation and detection of *Staphylococci* spp. to the selective Staphylococcus medium (Himedia, Mumbai, India) [40]. Pseudomonas agar medium (Himedia, Mumbai, India) was thus used for the identification of *Pseudomonas* species that displayed blue-green or brown pigmentation on medium after incubation for 24–48 h at 37 °C [41].

For *Salmonella* spp. detection, 25 g of the FFV sample was mixed with 100 mL of lactose (LB) broth, homogenized for 2 min, and incubated for 24–48 h at 37 °C. A total of 1 mL of the pre-enriched culture was then transferred to 10 mL of selenite broth and incubated for 24 h at 37 °C. On the bismuth sulfite agar (BSA) plate, the enriched culture was stripped and incubated for 24 h at 37 °C. On the BSA plate, standard *Salmonella* colonies were brown or gray to black colonies, often with a metallic shine. With little to no darkening of the surrounding medium, some strains create green colonies. For the biochemical test, two or more suspect colonies were selected from the BSA plate and streaked on the slants of triple sugar iron (TSI) agar. The tube was incubated for 24 h at 37 °C. Salmonella suspected cultures of TSI display red slants and yellow butts with or without H_2_S development (blackening of the agar) [42].

For enumeration of fungi, we spread 0.2 mL of first dilution (10^−1^ dilution) of each sample homogenate on five Petri dishes containing potato dextrose agar (PDA). The sample was homogeneously distributed on the plate using a glass spreader in a backward and forward motion when rotating the plate. The plates were then sealed and dried for 1–2 h before inverting. For 48–72 h, at room temperature, the prepared dishes were incubated. The plates were screened for the presence of discrete colonies after the incubation time. All colonies were counted on the plates containing colonies by a colony counter (Yc-2A, prma optical works Ltd., Tokushima; Japan) [43].

### 2.4. Preparation of Low-Cost Disinfectant Solution and Examination of the Washing Effect

Four types of disinfectant were used in this study, namely, distilled water, vinegar, salt water, and blanched. In a laminar flow biosafety cabinet, the whole fruit and salad vegetables were placed on a sterile surface. Both fruit and salads were cut to pieces aseptically (around 5 by 5 cm). In a 500 mL volumetric flask or beaker, the required quantity of decontaminated agents was tested. In a 500 mL volumetric flask or beaker, the selected 20 g sample was rinsed thoroughly with 180 mL of the various decontaminated solutions and was immersed in a disinfectant solution. Without washing, a portion of each tested sample was counted. The dip sample was shaken several times in diluents with hand- gloves to ensure complete solution coverage and to be fully settled. The species were assumed to have been washed off and distributed in diluents. The total viable bacterial count (TVBC/g) in tested unwashed samples was identified only as being dipped into sterile ringer solution, but TVBC was first reported in treated samples with four low-cost disinfectants and then dipped into sterile ringer solution and identified. The fruit and vegetable samples were dipped in sterile ringer solution (1:10) and plated for bacterial load enumeration with washed and unwashed samples. A 0.1 mL solution was taken from each of them and inoculated to count the microbes in the corresponding selective media. The FFV samples were washed for three minutes with distilled water, blanching (80–100 °C) for 1 min, vinegar (4.5 percent acetic acid) for a few seconds, and salt solutions (0.09 percent NaCl or 900 ppm) for 3 min to assess the effect of washing on the microbial load. Then, CFU/g enumerated the microbial load of the samples [44,45].

### 2.5. Statistical Analysis

All experiments were replicated three times. Total viable bacterial count (TVBC) was enumerated, and the microbial counts were expressed as log CFU/g. The log CFU/g reduction in bacterial population was calculated. The results of TVBC level from the surface of FFVs were analyzed for statistical significance using one-way analysis of variance (ANOVA) followed by LSD’s post hoc multiple comparison test using statistical software (SPSS) package version 21 (SPSS 21.0, US). *p* ≤ 0.05 was considered to be statistically significant.

## 3. Results

### 3.1. Viable Bacterial Load on Fresh Fruit and Vegetable Samples

Eighty-eight samples consisting of 12 types of fruit and 10 types of vegetable samples were tested for bacteriological and mycological load assessment. The viable bacterial ranges on FFV were different from sample types and collection places. In the case of fruit samples, the TVBC range was observed between 3.0 × 10^3^ (*P. embilica*) and 9.0 × 10^6^ CFU/g (*P. guajava*) in Jatrabari, 1.0 × 10^3^ (*B. ramiflora*) and 2.11 × 10^6^ CFU/g (*P. sylvestris*) in Mohammadpur, 1.0 × 10^3^ (*P. emblica*) and 3.2× 10^6^ CFU/g (*P. sylvestris*) in Uttara, and 7.0 × 10^2^ (*P. embilica*) and 2.81 × 10^6^ CFU/g (*P. sylvestris*) in Gulshan (Table S1). In the case of vegetable samples, the TVBC range was between 4.2 × 10^4^ (*C. sativus*) and 2.57 × 10^7^ CFU/g (*V.sinensis*) in Gulshan, between 5.9 × 10^4^ (*S. melongena*) and 2.6 × 10^7^ CFU/g (*V. sinensis*) in Mohammadpur, between 6.7 × 10^4^ (*S. melongena*) and 2.25 × 10^7^ CFU/g (*L. actangula*) in Uttara, and between 2.9 × 10^4^(*C. sativus*) and 2.17× 10^7^ CFU/g (*M. dioica*) in the sampling location of Jatrabari (Table S2).

Considering the tested fruit samples, the highest TVBC was detected at 6.47 ± 0.68 log CFU/g in the *P. guajava* sample, and the lowest bacterial count was found at 3.18 ± 0.27 log CFU/g in the *P. emblica* sample (Table 1).

On the other hand, the highest and lowest TVBCs were observed at 7.37 ± 0.06 log CFU/g (*V. sinensis*) and 5.67 ± 0.49 log CFU/g (*C. sativus*) on the surface of vegetable samples, respectively (Table 2). Among the fruit samples, the highest number of TCC was found (178.25 ± 10.46 MPN/g) in *S. dulcis*, and the highest TFC was found (1.92 ± 0.75 MPN/g) in *A. comosus* (Table 1). TFC was not detected in three samples, namely, apple (*M. pumila*), grape (*V. vinifera*), and lemon (*C. lemon*). On the surface of vegetables, the highest number of TCC was 56.5 ± 37.14 MPN/g in cucumber (*C. sativus*) and the highest number of TFC was 3.78 ± 0.76 MPN/g in teasle gourd (*M. dioica)* (Table 1). The presence of TFC was about 100% (40/40) in the vegetable sample and 88.33% (40/48) in the fruit sample (Table 1 and Table 2).

*Salmonella* was not present in any fruit sample (Table 1), but they were detected 15.0% (6/40) in vegetable samples (Table 2). Moreover, *Staphylococcus*, *Pseudomonas*, and fungi were frequently isolated in all vegetable and fruit samples. In case of fruit, the highest number of *Staphylococcus*, *Pseudomonas*, and fungi were detected in 5.10 ± 0.02, 5.38 ± 0.08, and 7.50 ± 0.15 log CFU/g in *P. sylvestris* and *C. grandis,* respectively (Table 1). Similarly, in case of the vegetable sample, the highest levels of *Staphylococcus*, *Pseudomonas*, and fungi were detected at 4.81 ± 0.16, 4.75 ± 0.23, and 4.40 ± 0.38 log CFU/g in *V. sinensis*, *T. dioeca*, and *M. charantia*, respectively (Table 2).

### 3.2. Washing Effect of Different Low-Cost Disinfectant Solutions on Fresh Fruits and Vegetables

**Figure 2:**
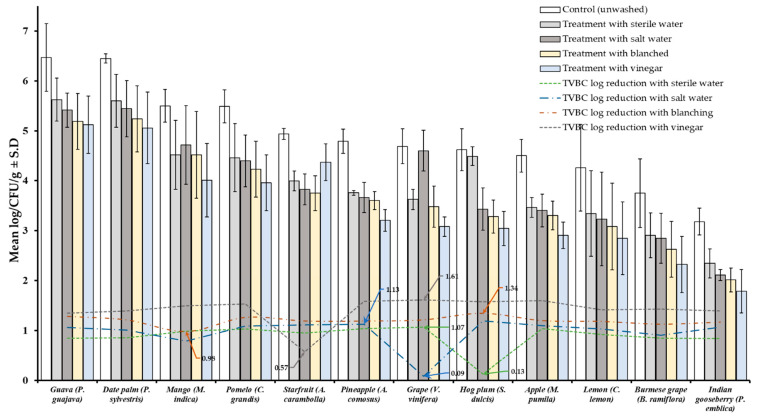
Four different treatments (sterile water, salt solution, blanched, and vinegar) were observed on total viable bacterial load (TVBC) decontamination from the surface of 12 different types of fruit. Data are mean (*n* = 4) ± standard deviation.

**Figure 3:**
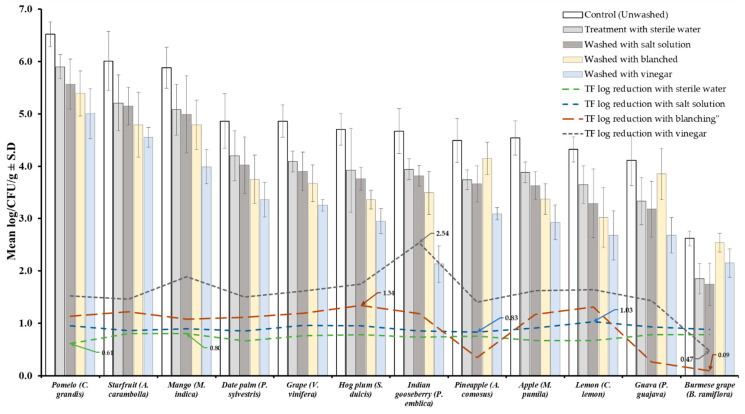
Four different treatments (sterile water, salt solution, blanched, and vinegar) were observed on total fungi load (TF) decontamination from the surface of 12 different types of fruit. Data are mean (*n* = 4) ± standard deviation.

**Figure 4:**
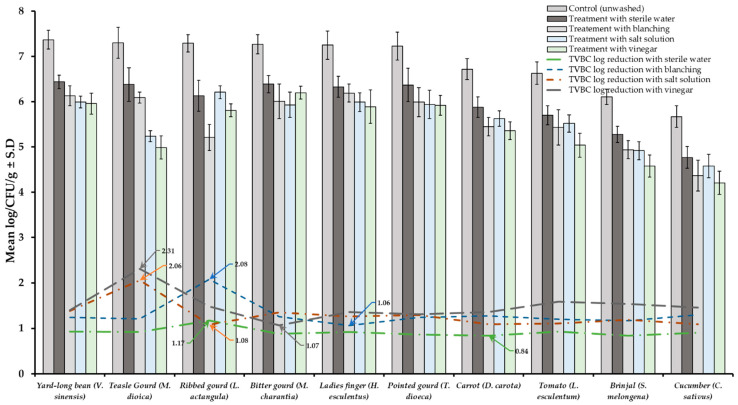
Four different treatments (sterile water, salt solution, blanched, and vinegar) were observed on total viable bacterial load (TVBC) decontamination from the surface of 10 different types of vegetables. Data are mean (*n* = 4) ± standard deviation.

**Figure 5:**
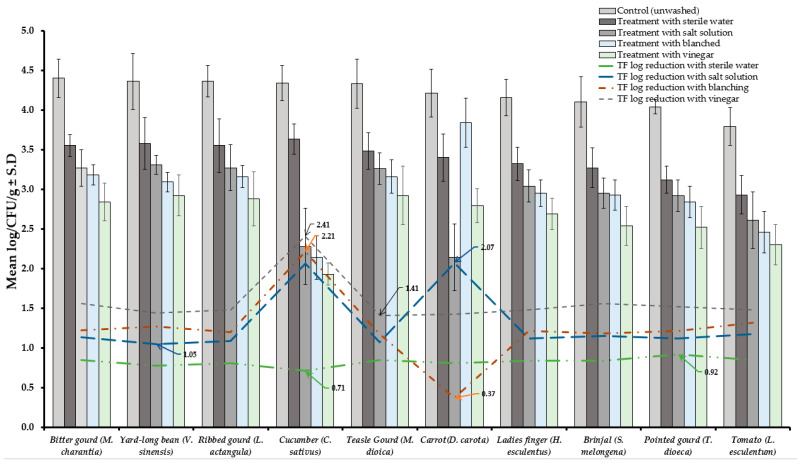
Four different treatments (sterile water, salt solution, blanched and vinegar) were observed on fungi load (TF) decontamination from the surface of 10 different types of vegetables. Data are mean (*n* = 4) ± standard deviation.

Results of TVBC and total fungi decontamination of fruit and vegetables are presented in Figure 2, Figure 3, Figure 4 and Figure 5. Among the four treatments, the vinegar solution showed the highest efficiency for TVBC and total fungi reduction on FFV samples. The initial populations of TVBC range was 3.18 ± 0.27 (*P. emblica*) and 6.47 ± 0.68 (*P*. *guajava*) and the total fungi range was 2.60 ± 0.18 (*B. ramiflora*) and 7.50 ± 0.15 (*C. grandis*) log CFU/g on the unwashed fruit samples, respectively (Table 1 and Table 2, Figure 2 and Figure 3). On vegetable samples, TVBC range was 5.67 ± 0.49 (*C. sativus*) and 7.37 ± 0.06 (*V. sinensis*) and the total fungi range was 3.79 ± 0.18 (*L. esculentum*) and 4.40 ± 0.38 (*M. charantia*) log CFU/g. On the basis of the treatments, we obtained a greater result of microbial decontamination. Three was a decreasing trend or pattern compared to control (vinegar > blanching > salt solution > sterile water > control) of TVBC and TF reduction from the surface of fruit samples (Figure 2 and Figure 3). Similarly decreasing trend was observed in TF reduction except *D. carota*, but decreasing trend was not observed in TVBC reduction (Figure 4 and Figure 5) With the treatment of sterile water on the FFV samples for 3 min, the TVBC reduction range was 0.13 to 1.07-log and the fungi reduction range was 0.61 to 0.80-log CFU/g (Figure 2 and Figure 3). When organic solution vinegar was used to wash fruits for a few seconds, the reduction of TBVC and TF range was 1.61 and 2.54 log CFU/g, respectively, higher than those observed with sterile water, blanched, and salt solution on the tested fruit samples (Figure 2 and Figure 3). In the case of TVBC and TF, the treatment of vinegar solution was significantly (*p* > 0.05) higher compared to control (unwashed treatment), and this treatment was also more efficient than the sterile water, blanched, and salt solution treatments.

Similarly, the highest reduction of TVBC on the surface of vegetables was 2.31-log with vinegar treatment and the following highest 2.08, 2.06, and 1.17-log reduction of TVBC with the treatment of blanching, salt solution, and being washed with sterile water, respectively (Figure 4). With the treatment of vinegar solution, we achieved the highest (2.41 log) fungi load reduction, as well as the following highest 2.21, 2.07, and 0.92-log TF reductions with the treatment of blanching, salt solution, and washing with sterile water, respectively, on the surface of vegetable samples (Figure 5).

## 4. Discussion

The present study evaluated the total microbial load on fresh fruits and vegetables (FFV) and concentrated on the safety of FFV by eliminating microorganisms. The indicator bacteria (TCC and TFC), the total viable bacteria (TVBC), and the total presence of fungi on the FFV surface reflect the sanitary nature of Agricultural products [34,35,36]. The isolated bacterial strains with higher TVBC were observed in studied fruits and vegetables (Table 1 and Table 2), and similar findings were also obtained in other studies [46,47]. However, the isolated bacterial counts reported in this study exceeded the recommended levels according to the International Commission on Microbiological Specifications for Foods [48]. The reference value of per gram FFV sample was 10-100 coliforms CFU/g, 10 fecal coliforms CFU/g, and 4.9 × 10^6^ total viable bacterial count CFU/g. The TVBC on fresh fruit may range from 10^2^ to 10^6^ CFU/g, depending on samples, locations, and conditions. Furthermore, the total count of fungi on fresh fruit and vegetables may vary from 10^3^ to 10^4^ CFU/g [49]. *Staphylococcus* spp. relies on various fruit and vegetables ranging from 10^2^ to 10^3^ CFU/g, and *Pseudomonas* spp. relies on products ranging from <10^2^ to 10^4^ which are sufficient in their ability to cause illness [50].

In this study, high microbial contamination was observed on the surface of FFV, which may reflect non-hygienic food handling practices, poor storage conditions, and poor sample processing and selling practices. The presence of fecal coliform as an indicator organism suggesting fecal contamination of tested fruits [51,52]. The fecal coliform was presented in our results with other bacteria such as *Pseudomonas*, and *Staphylococcus* was also observed in the FFV samples. The researchers also observed the presence of *Salmonella* spp., *Pseudomonas aeruginosa*, and *Staphylococcus aureus* in vegetables and fruits [36,53,54]. These findings indicate that we should clean these vegetables properly before consumption. The overall prevalence of *Salmonella* species in vegetables was also found to be 8% (*n* = 72) [36,54], which was close to our result (Table 2). Moreover, similar findings were recorded in a study on fresh fruit juices in Dhaka City, Bangladesh [55]. In another report, in Mymensingh District, Bangladesh, a total of 25 fresh-cut guava samples were collected, and a TVBC range of 6.47–6.62 log CFU/mL was detected in the fruit samples [34]. Similarly, the average TVBC range was 6.47 log CFU/g in our study. Nawas et al. (2012) conducted research of 15 (*n* = 15) salad vegetables collected from restaurants of different areas of Chittagong City, Bangladesh. In this research, they detected the total coliform (TC) around 73.33% (11/15) in salad samples, whereas 100% (40/40) vegetables samples were detected in our study. According to the report of Oranusi and Olorunfemi (2011), the mean TVBC range from 4.3–8.9 log CFU/g was obtained in pineapple and watermelon [56], and this result was also similar to our observation (Table 2). It was observed in a study of citrus fruit (lemon, lotkone, orange, malta, and amoloki) that the highest fungal count was 4.6 log CFU/g, and the highest TVBC count was 5.3 log CFU/g [57], which was similar to our study. In addition to our study, the average fungi ranges were between 3.57 and 4.40 log CFU/g in vegetables (Table 1) and 2.62 and 6.52 log CFU/g in fruit (Table 2).

The mean TVBC ranged from 5.67 log CFU/g to 7.37 CFU/g, indicating the presence of relatively higher bacterial presence in vegetable samples as per the recommended value of the International Commission on Microbiological Specifications for Foods (ICMSF). The presence of high microbial contamination associated with FFV samples indicated that the overall poor sanitary conditions and personal hygiene and sale practices are very poor in Dhaka City. Therefore, there is an unmet need for proper treatment to reduce microbial contamination from the FFV samples.

Washing is an important step for the decontamination of microbes of any fresh fruits and vegetables in postharvest processing. The use of running tap water to clean FFV samples before consumption is a traditional method that has been used for centuries. However, washing FFVs using tap water can cause cross-contamination, or it does not play a significant role in microbial reduction [58]. Results from the present study ensure the significance of using decontaminating agents when washing fresh produce such as fruits and vegetables. In our study, among the different treatments, vinegar showed the highest microbial load reduction of tested fruits and salad vegetables, whereas washing with sterile water showed the lowest microbial reduction (Figure 2, Figure 3, Figure 4 and Figure 5).

It has been observed that inoculated *Salmonella* and *E. coli* were decreased by 0.5 log 10 with rinsing water on the apple surface [59]. Tango et al. (2017) used whole apple and tomato fruits with inoculated and un-inoculated cocktail strains of *E. coli* O157:H7 and *Listeria monocytogenes*. Inoculated fruits were washed first with distilled water for 3 min [58] where <1 log 10 reduction of inoculated fruits surface bacteria, which is similar to our result (Tables S1 and S2). Besides this, using deionized water on oranges for 8 min achieved 2 log reduction of a mixture of *E. coli* strains [60]. It has been reported that blanching as a physical method can eliminate microbes from the surface of FFVs, owing to the thermal effect, resulting in the inactivation of enzymes [44]. In our study, the TVBC (0.98–1.34 log) and TF (0.09–1.34 log) reduction were observed due to hot water treatment (blanching) (Figure 2 and Figure 3).

Interestingly, with the help of chlorine-based solution treatment, the pathogenic bacteria introduced on tomatoes were reduced by more than 3.0 log [10]. A previous study showed that washing with chlorine solution could effectively reduce microbial populations by 10-100-fold [61]. In our study, the highest TVBC and fungi load reduction was found >2.0 log on the surface of the vegetables sample sodium chloride solutions for 3 min (Figure 4 and Figure 5). It has been reported that the efficacy of the decontaminating agents was highly influenced by the suitable concentration of chlorine [62]. Doyle and Erickson (2008) observed the naturally contaminated FFVs of the bacterial population were reduced 1–2 log CFU/g with the sanitizer or decontaminating agents [63]. Microbial load reduction was observed in fruits and vegetables washed in a vinegar solution [64,65,66]. Organic acids, especially vinegar (acetic acid), generally recognized as safe by the FDA and European Commission, are being well accepted by consumers as antimicrobial agents that are also considered to have great potential to control a wide range of microorganisms [67,68]. All these low-cost disinfectants are the emerging eco-friendly technique for preserving the quality and safety of fresh products.

## 5. Conclusions

The high microbial contamination rates associated with fresh fruits and vegetables indicate that the overall quality of fresh produce is poor enough from the microbiological point of view and the standard criteria determined by the Regulatory and International food safety agencies. This study explored the efficacy of the low-cost disinfectants such as distilled water, vinegar solution, chlorine solution, and hot water treatments in the decontamination of commonly consumed FFVs. Among them, low-cost sodium chloride (salt) solution or vinegar can be used as alternative decontaminating agents to reduce the load on surface microbes of fresh fruits and leafy vegetables. This study also provides valuable food safety information about the pathogens and indicator organisms that could be used to establish preventive measures to improve or ensure the quality and safety of fresh produce. In conclusion, an accurate, easy handle, and low-cost approach of disinfectant (sodium chloride-based solution salt) can be used for reducing the risk of contamination of fresh fruits and vegetables. Further research could be investigated on a variety of fruits and vegetables samples in order to find a standardized, more effective, and efficient sanitization approach.

## Figures and Tables

**Table 1:** Microbial assessment result of fresh cut fruit from different outlets in Dhaka City.

Tested Samples	Average Log CFU/g ± S.D				Presence/Absence	Average MPN/g ± SD	
	TVBC	*Staphylococci*	*Pseudomonas*	Total Fungi	*Salmonella*/25 g	TCC	TFC
Guava	^a^ 6.47 ± 0.68	^abc^ 4.39 ± 0.96	^b^ 4.67 ± 0.45	^g^ 4.10 ± 0.08	×	^abc^ 113.75 ± 11.04	^bc^ 0.56 ± 0.48
Date palm	^a^ 6.45 ± 0.09	^a^ 5.10 ± 0.02	^a^ 5.38 ± 0.08	^cd^ 4.86 ± 0.03	×	^abc^ 75.00 ± 8.26	^bc^ 0.56 ± 0.48
Mango	^b^ 5.50 ± 0.33	^cdef^ 3.62 ± 0.25	^cd^ 3.51 ± 0.09	^cdef^ 4.51 ± 0.25	×	^ab^ 138.25 ± 24.50	^b^ 0.98 ± 0.21
Pomelo	^bc^ 5.49 ± 0.33	^cde^ 3.94 ± 0.34	^b^ 4.84 ± 0.55	^a^ 7.50 ± 0.15	×	^a^ 175.00 ± 11.31	^bc^ 0.28 ± 0.25
Starfruit	^bcd^ 4.94 ± 0.11	^b^ 4.97 ± 0.04	^ef^ 2.79 ± 0.07	^b^ 6.00 ± 0.11	×	^abc^ 118.00 ± 11.20	^bc^ 0.28 ± 0.25
Pineapple	^bcd^ 4.79 ± 0.24	^def^ 3.31 ± 0.33	^de^ 3.22 ± 0.34	^ef^ 4.47 ± 0.12	×	^abc^ 99.00 ± 17.72	^a^ 1.92 ± 0.75
Grape	^cd^ 4.69 ± 0.35	^def^ 3.35 ± 0.99	^de^ 3.15 ± 0.56	^c^ 4.80 ± 0.14	×	^bc^ 37.20 ± 10.50	×
Hog plum	^de^ 4.62 ± 0.42	^efg^ 3.09 ± 0.74	^g^ 1.88 ± 0.03	^cde^ 4.69 ± 0.14	×	^a^ 178.25 ± 10.46	^bc^ 0.28 ± 0.56
Apple	^de^ 4.45 ± 0.33	^bcd^ 4.12 ± 0.78	^cd^ 3.51 ± 0.09	^cdef^ 4.50 ± 0.25	×	^bc^ 22.10 ± 9.31	×
Lemon	^ef^ 4.26 ± 0.87	^fgh^ 2.83 ± 0.51	^c^ 3.84 ± 0.58	^fg^ 4.29 ± 0.30	×	^ab^ 150.5 ± 110.81	×
Burmese grape	^fg^ 3.75 ± 0.69	^gh^ 2.42 ± 0.11	^f^ 2.59 ± 0.24	^h^ 2.60 ± 0.18	×	^abc^ 104.25 ± 10.31	^bc^ 0.42 ± 0.21
Indian gooseberry	^g^ 3.18 ± 0.27	^h^ 2.04 ± 0.53	^fg^ 2.29 ± 0.35	^cdef^ 4.56 ± 0.38	×	^abc^ 62.58 ± 10.31	^b^ 0.88 ± 0.31

Mean values followed by different letters within a column of tested samples were found to be significantly different using a post hoc multiple comparisons test (*p* < 0.05). MPN = most probable number; CFU = colony-forming unit; TVBC = total viable bacterial count; TCC = total coliform count; TFC = total fecal coliform; “×” = not detected. Data are mean (*n* = 4) ± standard deviation.

**Table 2:** Microbial assessment of fresh vegetables collected from different urban areas in Dhaka City.

Samples	Average Log CFU/g ± S.D				Presence/Absence	Average MPN/g ± S.D	
	TVBC	*Staphylococci*	*Pseudomonas*	Total Fungi	*Salmonella*/25 g	TCC	TFC
Yard-long bean	^a^ 7.37 ± 0.06	^a^ 4.81 ± 0.16	^bc^ 4.31 ± 0.23	^a^ 4.36 ± 0.11	0% (0/4)	^abc^ 28.75 ± 11.59	^ab^ 1.76 ± 0.87
Teasle gourd	^a^ 7.30 ± 0.04	^a^ 4.77 ± 0.25	^ab^ 4.65 ± 0.24	^a^ 4.33 ± 0.32	25% (1/4)	^ab^ 51.00 ± 40.67	^ab^ 3.78 ± 3.76
Ribbed gourd	^a^ 7.29 ± 0.09	^a^ 4.79 ± 0.15	^ab^ 4.52 ± 0.17	^a^ 4.36 ± 0.21	50% (2/4)	^abc^ 18.45 ± 18.96	^ab^ 1.76 ± 0.87
Bitter gourd	^a^ 7.27 ± 0.15	^a^ 4.77 ± 0.27	^ab^ 4.56 ± 0.44	^a^ 4.40 ± 0.38	25% (1/4)	^ab^ 51.00 ± 40.68	^ab^ 1.76 ± 0.87
Ladies finger	^a^ 7.25 ± 0.19	^a^ 4.51 ± 0.56	^ab^ 4.69 ± 0.23	^a^ 4.16 ± 0.19	50% (2/4)	^abc^ 44.75 ± 43.53	^ab^ 1.76 ± 0.87
Pointed gourd	^a^ 7.23 ± 0.21	^a^ 4.73 ± 0.15	^a^ 4.75 ± 0.23	^ab^ 4.04 ± 0.08	0% (0/4)	^abc^ 28.75 ± 11.59	^a^ 1.76 ± 0.87
Carrot	^b^ 6.72 ± 0.89	^b^ 3.73 ± 0.10	^c^ 4.04 ± 0.34	^a^ 4.21 ± 0.08	0% (0/4)	^c^ 1.85 ± 1.11	^b^ 1.76 ± 0.87
Tomato	^bc^ 6.63 ± 0.84	^b^ 3.55 ± 0.16	^d^ 3.63 ± 0.20	^b^ 3.79 ± 0.18	0% (0/4)	^bc^ 6.08 ± 9.97	^b^ 1.76 ± 0.87
Brinjal	^cd^ 6.61 ± 0.52	^b^ 3.63 ± 0.28	^d^ 3.61 ± 0.24	^a^ 4.10 ± 0.18	0% (0/4)	^abc^ 21.57 ± 17.67	^ab^ 1.76 ± 0.87
Cucumber (*Cucumis sativus*)	^d^ 5.67 ± 0.49	^b^ 3.48 ± 0.13	^d^ 3.57 ± 0.21	^a^ 4.34 ± 0.07	0% (0/4)	^a^ 56.50 ± 37.14	^a^ 1.76 ± 0.87

Mean values followed by different letters within a column of tested samples were found to be significantly different using a post hoc multiple comparisons test (*p* < 0.05). MPN = most probable number; CFU = colony-forming unit; TVBC = total viable bacterial count; TCC = total coliform count; TFC = total fecal coliform; “×” = not detected. Data are mean (*n* = 4) ± standard deviation.

## Data Availability

All data are provided within this paper.

## Supplementary Materials

The following are available online at https://www.mdpi.com/article/10.3390/foods10061325/s1. Table S1: Total viable bacteria count (TVBC) of fresh fruits collected from different urban areas in Dhaka city. Table S2: Total viable bacteria count (TVBC) of fresh vegetables collected from different urban areas in Dhaka city.

## References

[1] United Nations (UN) (2011). World Urbanization Prospects, the 2011 Revision.

[2] Siddique M.M.A. (2017). Effect of Organic Farming on Growth, Yield and Quality of Lettuce and on Soil Properties. Master’s Thesis.

[3] Mahfuza I., Arzina H., Kamruzzaman M.M., Afifa K., Afzal H.M., Rashed N., Roksana H. (2016). Microbial status of street vended fresh-cut fruits, salad vegetables and juices in Dhaka city of Bangladesh. Int. Food Res. J..

[4] Slavin J.L., Lloyd B. (2012). Health benefits of fruits and vegetables. Adv. Nutr..

[5] Di Cagno R., Coda R., De Angelis M., Gobbetti M. (2013). Exploitation of vegetables and fruits through lactic acid fermentation. Food Microbiol..

[6] Boeing H., Bechthold A., Bub A., Ellinger S., Haller D., Kroke A., Leschik-Bonnet E., Müller M.J., Oberritter H., Schulze M. (2012). Critical review: Vegetables and fruit in the prevention of chronic diseases. Eur. J. Nutr..

[7] Christison C., Lindsay D., Von Holy A. (2008). Microbiological survey of ready-to-eat foods and associated preparation surfaces in retail delicatessens, Johannesburg, South Africa. Food Control.

[8] Losio M., Pavoni E., Bilei S., Bertasi B., Bove D., Capuano F., Farneti S., Blasi G., Comin D., Cardamone C. (2015). Microbiological survey of raw and ready-to-eat leafy green vegetables marketed in Italy. Int. J. Food Microbiol..

[9] Santos M.I., Correira C., Cunha M.I.C., Saraiva M.M., Novais M.R. (2005). Valores Guia para avaliação da qualidade microbiológica de alimentos prontos a comer preparados em estabelecimentos de restauração. Rev. Ordem Farm..

[10] Food Safety Authority of Ireland (FSAI) (2016). Guidance Note No. 3: Guidelines for the Interpretation of Results of Microbiological Testing of Ready-to-Eat Foods Placed on the Market (Revision 2).

[11] Food Standards Australia New Zealand (FSANZ) (2001). Microbiological Quality Guide for Ready-to-Eat Foods. A Guide to Interpreting Microbiological Results. https://www.foodauthority.nsw.gov.au/sites/default/files/Documents/scienceandtechnical/microbiological.

[12] Alberta Health Services (2011). Microbial Guidelines for Ready-to-Eat Foods. A Guide to Interpreting Microbiological Results. https://www.albertahealthservices.ca/assets/wf/eph/wf-eh-microbial-guidelines-for-ready-to-eat-foods.pdf.

[13] Tango C.N., Wei S., Khan I., Hussain M.S., Kounkeu P.F.N., Park J.H., Kim S.H., Oh D.H. (2018). Microbiological quality and safety of fresh fruits and vegetables at retail levels in Korea. J. Food Sci..

[14] Ngnitcho P.-F.K., Khan I., Tango C.N., Hussain M.S., Oh D.H. (2017). Inactivation of bacterial pathogens on lettuce, sprouts, and spinach using hurdle technology. Innov. Food Sci. Emerg. Technol..

[15] Rahman S., Khan I., Oh D.H. (2016). Electrolyzed water as a novel sanitizer in the food industry: Current trends and future perspectives. Compr. Rev. Food Sci. Food Saf..

[16] Razzaq R., Farzana K., Mahmood S., Murtaza G. (2014). Microbiological Analysis of Street Vended Vegetables in Multan City Pakistan: A Public Health Concern. Pak. J. Zool..

[17] Abadias M., Alegre I., Oliveira M., Altisent R., Viñas I. (2012). Growth potential of Escherichia coli O157: H7 on fresh-cut fruits (melon and pineapple) and vegetables (carrot and escarole) stored under different conditions. Food Control.

[18] Huang Y., Chen H. (2011). Effect of organic acids, hydrogen peroxide and mild heat on inactivation of *Escherichia coli* O157: H7 on baby spinach. Food Control.

[19] Buchholz U., Bernard H., Werber D., Böhmer M.M., Remschmidt C., Wilking H., Deleré Y., an der Heiden M., Adlhoch C., Dreesman J. (2011). German outbreak of Escherichia coli O104: H4 associated with sprouts. N. Engl. J. Med..

[20] Pan X., Nakano H. (2014). Effects of chlorine-based antimicrobial treatments on the microbiological qualities of selected leafy vegetables and wash water. J. Food Sci. Technol..

[21] Pollack S. (2001). Consumer Demand for Fruit and Vegetables: The U.S. Example. http://www.ers.usda.gov/webdocs40303/14977_wrs011h_1_.pdf?v=5008.3.

[22] Arienzo A., Murgia L., Fraudentali I., Gallo V., Angelini R., Antonini G. (2020). Microbiological Quality of Ready-to-Eat Leafy Green Salads during Shelf-Life and Home-Refrigeration. Foods.

[23] Gould L.H., Walsh K.A., Vieira A.R., Herman K., Williams I.T., Hall A.J., Cole D. (2013). Centers for Disease Control and Prevention. Surveillance for foodborne disease outbreaks—United States, 1998–2008. MMWR Surveill. Summ..

[24] Berger C.N., Sodha S.V., Shaw R.K., Griffin P.M., Pink D., Hand P., Frankel G. (2010). Fresh fruit and vegetables as vehicles for the transmission of human pathogens. Environ. Microbiol..

[25] Lynch M.F., Tauxe R.V., Hedberg C.W. (2009). The growing burden of foodborne outbreaks due to contaminated fresh produce: Risks and opportunities. Epidemiol. Infect..

[26] Little C.L., Gillespie I.A. (2008). Prepared salads and public health. J. Appl. Microbiol..

[27] Havelaar A.H., Kirk M.D., Torgerson P.R., Gibb H.J., Hald T., Lake R.J., Praet N., Bellinger D.C., De Silva N.R., Gargouri N. (2015). World Health Organization global estimates and regional comparisons of the burden of foodborne disease in 2010. PLoS Med..

[28] Qadri F., Svennerholm A.-M., Faruque A., Sack R.B. (2005). Enterotoxigenic Escherichia coli in developing countries: Epidemiology, microbiology, clinical features, treatment, and prevention. Clin. Microbiol. Rev..

[29] IEDCR (2018). Outbreak. https://www.iedcr.gov.bd/website/index.php/outbreak.

[30] Jasson V., Jacxsens L., Luning P., Rajkovic A., Uyttendaele M. (2010). Alternative microbial methods: An overview and selection criteria. Food Microbiol..

[31] Banach J., Van Bokhorst-van de Veen H., Van Overbeek L., Van der Zouwen P., Van der Fels-Klerx H., Groot M.N. (2017). The efficacy of chemical sanitizers on the reduction of Salmonella Typhimurium and Escherichia coli affected by bacterial cell history and water quality. Food Control.

[32] Parish M., Beuchat L., Suslow T., Harris L., Garrett E., Farber J., Busta F. (2003). Methods to reduce/eliminate pathogens from fresh and fresh-cut produce. Compr. Rev. Food Sci. Food Saf..

[33] Sapers G., Miller R., Annous B., Burke A. (2002). Improved antimicrobial wash treatments for decontamination of apples. J. Food Sci..

[34] Sarker M.A.R., Haque M.M., Rifa R.A., Ema F.A., Islam M.A., Khatun M.M. (2018). Isolation and identification of bacteria from fresh guava (Psidium guajava) sold at local markets in Mymensingh and their antibiogram profile. Vet. World.

[35] Feroz F., Noor R. (2018). Transmission of pathogens within the commonly consumed vegetables: Bangladesh perspective. Stamford J. Microbiol..

[36] Nawas T., Mazumdar R., Das S., Nipa M., Islam S., Bhuiyan H., Ahmad I. (2012). Microbiological quality and antibiogram of *E. coli*, Salmonella and Vibrio of salad and water from restaurants of Chittagong. J. Environ. Sci. Nat. Resour..

[37] Mohammadzadeh-Vazifeh M.M., Hosseini S.M., Khajeh-Nasiri S., Hashemi S., Fakhari J. (2015). Isolation and identification of bacteria from paperboard food packaging. Iran. J. Microbiol..

[38] Doğan-Halkman H.B., Çakır İ., Keven F., Worobo R.W., Halkman A.K. (2003). Relationship among fecal coliforms and Escherichia coli in various foods. Eur. Food Res. Technol..

[39] Kechero F.K., Baye K., Tefera A.T., Tessema T.S. (2019). Bacteriological quality of commonly consumed fruit juices and vegetable salads sold in some fruit juice houses in Addis Ababa, Ethiopia. J. Food Saf..

[40] Perry J.D., Rennison C., Butterworth L.A., Hopley A.L., Gould F.K. (2003). Evaluation of S. aureus ID, a new chromogenic agar medium for detection of Staphylococcus aureus. J. Clin. Microbiol..

[41] Yilmaz A.G. (2017). Development of a New Pseudomonas Agar Medium Containing Benzalkonium Chloride in Cetrimide Agar. Food Nutr. Sci..

[42] Kar J., Barman T.R., Sen A., Nath S.K. (2017). Isolation and identification of Escherichia coli and Salmonella sp. from apparently healthy Turkey. Int. J. Adv. Res. Biol. Sci..

[43] Delfiyana M., Umar S., Ginting N. (2018). Isolation and Characteristics of Corn-Based Cellulolytic Fungi as Fibrous Feed Bioactivators. J. Peternak. Integr..

[44] Bhilwadikar T., Pounraj S., Manivannan S., Rastogi N., Negi P. (2019). Decontamination of microorganisms and pesticides from fresh fruits and vegetables: A comprehensive review from common household processes to modern techniques. Compr. Rev. Food Sci. Food Saf..

[45] De Corato U. (2020). Improving the shelf-life and quality of fresh and minimally-processed fruits and vegetables for a modern food industry: A comprehensive critical review from the traditional technologies into the most promising advancements. Crit. Rev. Food Sci. Nutr..

[46] Tambekar D., Mundhada R. (2006). Bacteriological quality of salad vegetables sold in Amravati City (India). J. Biol. Sci..

[47] Bukar A., Uba A., Oyeyi T. (2010). Antimicrobial profile of Moringa oleifera Lam. extracts against some food–borne microorganisms. Bayero J. Pure Appl. Sci..

[48] ICMF (1998). Potential application of risk assessment techniques to microbiological issues related to international trade in food and food products. J. Food Prot..

[49] Webb T., Mundt J. (1978). Molds on vegetables at the time of harvest [Fungal populations, post-harvest treatments]. Appl. Environ. Microbiol. (USA).

[50] Prokopowich D., Blank G. (1991). Microbiological evaluation of vegetable sprouts and seeds. J. Food Prot..

[51] Montville T.J., Matthews K.R. (2013). Physiology, growth, and inhibition of microbes in foods. Food Microbiology.

[52] Abadias M., Usall J., Anguera M., Solsona C., Viñas I. (2008). Microbiological quality of fresh, minimally-processed fruit and vegetables, and sprouts from retail establishments. Int. J. Food Microbiol..

[53] Ibeyessie J. (2007). Bacterial pathogens recovered from vegetables irrigated by waste water. J. Environ. Health.

[54] Razzaq K., Khan A.S., Malik A.U., Shahid M., Ullah S. (2015). Effect of oxalic acid application on Samar Bahisht Chaunsa mango during ripening and postharvest. LWT Food Sci. Technol..

[55] Ahmed M.S.U., Nasreen T., Feroza B., Parveen S. (2009). Microbiological quality of local market vended freshly squeezed fruit juices in Dhaka city, Bangladesh. Bangladesh J. Sci. Ind. Res..

[56] Oranusi S., Olorunfemi O. (2011). Microbiological safety evaluation of street vended ready-to-eat fruits sold in Ota, Ogun state, Nigeria. Int. J. Res. Biol. Sci..

[57] Al Mamun S., Feroz F. (2017). Complete microbiological analysis of citrus fruits and the effect of heat on microbial load & antimicrobial activity. Stamford J. Microbiol..

[58] Tango C.N., Khan I., Kounkeu P.-F.N., Momna R., Hussain M.S., Oh D.-H. (2017). Slightly acidic electrolyzed water combined with chemical and physical treatments to decontaminate bacteria on fresh fruits. Food Microbiol..

[59] Beuchat L.R. (1998). Surface Decontamination of Fruits and Vegetables Eaten Raw: A Review.

[60] Artés-Hernández F., Martínez-Hernández G.B., Aguayo E., Gómez P.A., Artés F. (2017). Fresh-cut fruit and vegetables: Emerging eco-friendly techniques for sanitation and preserving safety. Postharvest Handl..

[61] Buck J., Walcott R., Beuchat L. (2003). Recent trends in microbiological safety of fruits and vegetables. Plant health progress.

[62] Ignat A., Manzocco L., Maifreni M., Nicoli M.C. (2016). Decontamination Efficacy of Neutral and Acidic Electrolyzed Water in Fresh-Cut Salad Washing. J. Food Process. Preserv..

[63] Doyle M.P., Erickson M.C. (2008). Summer meeting 2007—The problems with fresh produce: An overview. J. Appl. Microbiol..

[64] Baldas B., Altuner E.M. (2018). The antimicrobial activity of apple cider vinegar and grape vinegar, which are used as a traditional surface disinfectant for fruits and vegetables. Commun. Fac. Sci. Univ. Ank. Ser. C Biol..

[65] Vijayakumar C., Wolf-Hall C.E. (2002). Evaluation of household sanitizers for reducing levels of Escherichia coli on iceberg lettuce. J. Food Prot..

[66] Medina E., Romero C., Brenes M., de CASTRO A. (2007). Antimicrobial activity of olive oil, vinegar, and various beverages against foodborne pathogens. J. Food Prot..

[67] Warriner K., Huber A., Namvar A., Fan W., Dunfield K. (2009). Recent advances in the microbial safety of fresh fruits and vegetables. Adv. Food Nutr. Res..

[68] Giannakourou M.C., Tsironi T.N. (2021). Application of Processing and Packaging Hurdles for Fresh-Cut Fruits and Vegetables Preservation. Foods.

