# Supplementary material for: Microbial Quality Assessment and Efficacy of Low-Cost Disinfectants on Fresh Fruits and Vegetables Collected from Urban Areas of Dhaka, Bangladesh
Source: Foods. 2021 Jun 9;10(6):1325. doi: 10.3390/foods10061325 (PMC8227336; doi:10.3390/foods10061325)
Supplement: Supplementary file 1 [file foods-10-01325-s001.zip › foods-1240146-supplementary.pdf]

foods MDPI

Table S1. Total viable bacteria count (TVBC) of fresh fruits collected from different urban areas in Dhaka city.

Name of Sample fruits	Gulshan	Mohammadpur	Uttra	Jatrabari	Mean (± SD)CFU/g
Guava (*P. guajava*)	4.30×10^5	2.0×10^6	3.0×10^5	9.0×10^6	$2.93(\pm 4.12) \times 10^6$
Date palm (*P. sylvestris*)	2.81×10^6	2.11×10^6	3.20×10^6	3.31×10^6	$2.86(\pm 0.47) \times 10^6$
Mango (*M. indica*)	2.0×10^5	1.14×10^5	2.5×10^5	7.1×10^5	$3.19(\pm 2.67) \times 10^5$
Pomelo (*C. grandis*)	6.5×10^4	8.0×10^5	1.3×10^5	2.4×10^5	$3.09(\pm 3.35) \times 10^5$
Starfruit (*A. carambolla*)	5.9×10^4	8.9×10^4	9.9×10^4	10.4×10^4	$8.78(\pm 1.75) \times 10^4$
Pineapple (*A. comosus*)	2.7×10^4	9.1×10^4	7.8×10^4	5.6×10^4	$6.3(\pm 2.80) \times 10^4$
Grape (*V. vinifera*)	2.0×10^4	5.8×10^4	2.0×10^4	9.8×10^4	$4.9(\pm 3.72) \times 10^4$
Hog plum (*S. dulcis*)	1.0×10^4	5.0×10^4	2.0×10^4	9.0×10^4	$4.25(\pm 3.59) \times 10^4$
Apple (*M. pumila*)	2.0×10^4	1.14×10^4	2.5×10^4	7.1×10^4	$3.19(\pm 2.67) \times 10^4$
Lemon (*C. lemon*)	2.0×10^3	4.0×10^4	3.0×10^4	7.0×10^3	$1.98(\pm 1.82) \times 10^4$
Burmese grape (*B. ramiflora*)	5.0×10^3	1.0×10^3	9.0×10^3	1.2×10^4	$6.75(\pm 0.48) \times 10^3$
Indian gooseberry (*P. emblica*)	7.0×10^2	1.3×10^3	1.0×10^3	3.0×10^3	$1.50(\pm 1.03) \times 10^3$

Table S2. Total viable bacteria count (TVBC) of fresh vegetables collected from different urban areas in Dhaka city.

Name of vegetabl samples	Gulshan	Mohammadpur	Uttara	Jatrabari	Mean (± SD)CFU/g
Yard-long bean (*V. sinensis*)	2.57×10^7	2.62×10^7	2.23×10^7	1.95×10^7	$2.34(\pm 0.31) \times 10^7$
Teasle Gourd (*M. dioica*)	2.10×10^7	1.83×10^7	1.91×10^7	2.17×10^7	$2.00(\pm 0.16) \times 10^7$
Ribbed gourd (*L. actangula*)	2.22×10^7	1.96×10^7	2.25×10^7	1.44×10^7	$1.97(\pm 0.38) \times 10^7$
Bitter gourd (*M. charantia*)	2.45×10^7	1.87×10^7	1.12×10^7	2.04×10^7	$1.87(\pm 0.56) \times 10^7$
Ladies finger (*H. esculentus*)	2.10×10^7	8.70×10^6	2.19×10^7	1.91×10^7	$1.77(\pm 0.61) \times 10^7$
Pointed gourd (*T. dioeca*)	2.45×10^7	1.12×10^7	2.23×10^7	9.6×10^6	$1.69(\pm 0.76) \times 10^7$
Carrot (*D. carota*)	2.20×10^5	4.3×10^5	8.20×10^6	1.22×10^7	$5.30(\pm 5.9) \times 10^6$
Tomato (*L. esculentum*)	5.00×10^6	1.15×10^7	3.80×10^5	2.2×10^5	$4.30(\pm 5.3) \times 10^6$
Brinjal (*S. melongena*)	7.90×10^5	5.9×10^4	6.70×10^4	3.5×10^5	$4.67(\pm 3.3) \times 10^5$
Cucumber (*C. sativus*)	4.20×10^4	3.6×10^5	8.70×10^4	2.9×10^4	$1.3(\pm 1.56) \times 10^5$
